# Vacant Parking Slot Detection in the Around View Image Based on Deep Learning

**DOI:** 10.3390/s20072138

**Published:** 2020-04-10

**Authors:** Wei Li, Libo Cao, Lingbo Yan, Chaohui Li, Xiexing Feng, Peijie Zhao

**Affiliations:** 1 State Key Laboratory of Advanced Design and Manufacturing for Vehicle Body, Hunan University, Changsha 410006, China; lw_hnu@hnu.edu.cn (W.L.); hdclb@163.com (L.C.); hndxlch@hnu.edu.cn (C.L.); jeremyfeng@hnu.edu.cn (X.F.); 2 GAC Parts Corporation Limited, Guangzhou 510630, China; zhaopeijie@gacc.com.cn

**Keywords:** park assist system, vacant parking slot detection, deep learning, around view image

## Abstract

Due to the complex visual environment, such as lighting variations, shadows, and limitations of vision, the accuracy of vacant parking slot detection for the park assist system (PAS) with a standalone around view monitor (AVM) needs to be improved. To address this problem, we propose a vacant parking slot detection method based on deep learning, namely VPS-Net. VPS-Net converts the vacant parking slot detection into a two-step problem, including parking slot detection and occupancy classification. In the parking slot detection stage, we propose a parking slot detection method based on YOLOv3, which combines the classification of the parking slot with the localization of marking points so that various parking slots can be directly inferred using geometric cues. In the occupancy classification stage, we design a customized network whose size of convolution kernel and number of layers are adjusted according to the characteristics of the parking slot. Experiments show that VPS-Net can detect various vacant parking slots with a precision rate of 99.63% and a recall rate of 99.31% in the ps2.0 dataset, and has a satisfying generalizability in the PSV dataset. By introducing a multi-object detection network and a classification network, VPS-Net can detect various vacant parking slots robustly.

## 1. Introduction

With the rapid development of society, passenger cars are becoming more and more popular in large cities, which makes it difficult to find a vacant parking slot. A study shows that over 50% of drivers are frustrated by looking for free parking space in traffic dense area [1]. Besides, in total car collisions, 23% of accidents happen in parking lots [2]. In this context, the park assist system (PAS) is a promising technology most drivers want to see, which is composed of three parts: object position designation, path planning, and parking guidance or path tracking. As the most important component of the PAS, the task of the object position designation is to detect a vacant parking slot accurately. The PAS can be divided into four categories based on the parking space detection method: free space-based [3,4,5,6,7], parking slot marking-based [8,9,10,11], interface-based [12,13,14], and infrastructure-based [15,16,17]. Compared with other methods, the parking slot marking-based approach can be applied in wider situations, since it does not depend on the existence of adjacent vehicles or extra communication equipment. Moreover, as people pay more attention to vehicle safety, myriads of vehicles are equipped with the around view monitor (AVM) [18], which provides 360${}^{\circ }$ surveillance around the vehicle. Therefore, the vacant parking slot detection in the around view image can make full use of the existing equipment on the vehicle.

In order to make vacant parking slot detection in the around view image meaningful and practical, it should satisfy the following conditions: recognizing various types of parking slots and being robust under the complex visual environment. To this aim, a series of marking point-based parking slot detection methods were proposed by Suhr [19,20,21,22]. These methods utilize designed features to detect marking points, which are easily affected by lighting variations. To detect marking points robustly, a method utilizing the deep convolutional neural network (DCNN) was proposed in [11]. Due to the powerful feature extraction ability of DCNN, this method significantly improves the accuracy of parking slot detection. However, it cannot classify the parking slot occupancy status and involves a few cumbersome steps to infer the complete parking slot. To complement this method, an end-to-end DCNN was proposed in [23] to perform automatic parking slot detection and classification simultaneously. However, this method is based on the Faster R-CNN baseline and it cannot meet the real-time requirements. Moreover, a few semantic segmentation-based methods were proposed in recent years, such as VH-HFCN [24] and DFNet [25]. Despite these methods having outstanding performance in ground markings segmentation, they need post-processing to generate parking slots, which is time-consuming and inaccurate. A detailed literature review will be presented in Section 2.

In view of the limitations of previous works, we attempt to devise a vacant parking slot detection method with a standalone AVM based on DCNN, namely VPS-Net, which can not only detect various types of vacant parking slots effectively but also meet real-time requirements. VPS-Net converts the vacant parking slot detection into a two-step problem, including parking slot detection and occupancy classification. In the parking slot detection process, we first detect and classify all marking points and parking slot heads using a pre-trained detector based on YOLOv3 [26]. Then, the geometric information is used to match paired marking points and infer the complete parking slot. In the occupancy classification process, a customized DCNN is designed to make the parking slot occupancy classification reliable. Finally, VPS-Net is evaluated in the ps2.0 dataset [11] and PSV dataset [24]. The results show that VPS-Net outperforms previous methods with a precision rate of 99.63% and a recall rate of 99.31%. Moreover, it achieves a real-time detection speed of 20.5 ms per frame on Nvidia Titan Xp.

The contributions of this paper are as follows:

- A new vacant parking slot detection method in the around view image is proposed, and we name it as VPS-Net, which combines the advantages of a multi-object detection network with a classification network. Compared with the semantic segmentation-based methods that need a series of complex post-processing to get the position of the parking slot, VPS-Net can directly get the coordinates of marking points, so the more accurate position of parking slots can be achieved. To facilitate future researchers, the related codes and the annotations for vacant parking slots of ps2.0 and PSV datasets have been made publicly available at https://github.com/weili1457355863/VPS-Net.
- A parking slot detection method based on YOLOv3 is proposed, which combines the classification of the parking slot with the localization of marking points. Compared with previous marking point-based methods that cumbersome steps are required to match the paired marking points of the parking slot, VPS-Net simplifies the process of parking slot detection, so various kinds of parking slots can be detected quickly and robustly.
- A customized DCNN model is designed to distinguish whether it is a vacant parking slot. To evaluate the performance of the model, we update both ps2.0 and PSV datasets by marking the type of parking slot in each image. Compared with some state-of-the-art (SOTA) DCNN models, our customized DCNN model not only achieves comparable accuracy but also consumes less time to process an image and has fewer parameters.

The remainder of this paper is organized as follows. Section 2 introduces the related research. Section 3 describes the details of the VPS-Net method. Section 4 presents the experimental results of the VPS-Net. Finally, the paper is discussed and concluded with a summary in Section 5 and Section 6.

## 2. Related Works

In this paper, our method mainly includes the detection of parking slots in the around view image and the classification of parking slot occupancy. Related works about these aspects will be described in detail here.

### 2.1. Vision-Based Parking Slot Detection in the Around View Image

In the past few years, various vision-based parking slot detection methods in the around view image have emerged. These approaches mainly could be subdivided into three categories: line-based methods [8,9,27,28], marking point-based methods [10,11,19,20,21,22], and segmentation-based methods [24,25,29]. Hamada et al. [27] used the Sobel filter and probabilistic Hough transform to obtain lines as the potential parking slot markers. However, the Hough transform is easily influenced by lighting conditions and strong shadows. To improve the noise-tolerance ability and robustness of parking slot detection, Wang et al. [28] proposed a new method based on Randon transform to detect straight lines and mitigate the effects of noises effectively through clustering and filtering according to the special shape characteristics of the parking slot. Lee et al. [8] designed a Directional-DBSCAN line-level feature-clustering algorithm to solve the short, distorted lines of the parking slot in around view images. After that, the decision-tree classifier was designed to determine the specific type of parking slot. Li et al. [9] combined line clustering with multi-view learning to detect the separating lines and entrance lines of parking slots, and then geometric features were utilized to recognize parking slots. However, these classical methods are based on primitive line features and are not robust to the real complicated environment.

Unlike these line-based methods, some parking slot detection methods are based on marking points detection. Suhr and Jung [19] took advantage of the Harris corner detector [30] to detect corners of parking slots and then generated junction candidates. At last, various types of parking slots can be predicted based on the characteristics of junction candidates. Zhang et al. [10] proposed a machine learning-based approach called PSD_L, which detected the marking-points using AdaBoost [31] framework first and then inferred complete parking-slots using detection results. To further improve the robustness of parking slot detection, Zhang et al. [11] then proposed a DCNN-based approach called DeepPS, which is the first work using deep learning techniques to detect parking slots. The YOLOv2 [32] detector was utilized to detect marking points first, and then a customized classification network was designed to obtain the orientation of the parking slot. Finally, the parking slot was inferred from detection results. This method is similar to VPS-Net, but it involves a few cumbersome steps to infer the complete parking slot and cannot classify parking slot occupancy.

In recent years, a few deep learning-based semantic segmentation methods have been proposed for improving the reliability of parking slots detection under harsh conditions. Jang and Sunwoo [29] proposed a semantic segmentation-based parking slot detection method. A semantic segmentation network was utilized to classify vehicles, free space, markings of the parking slot, and other objects. Furthermore, they proposed a vertical-grid-based parking slots refinement that provided refined parking slots information. To robustly and precisely extract linear features of parking slots, Wu et al. [24] proposed a VH-HFCN network, which adopted a highly fused convolutional network as the base and added VH-stage for better segmenting lines of the parking slot. The VH-stage was specially designed to extract linear features, containing independent horizontal and vertical convolution kernels. Subsequently, Jiang et al. [25] proposed a DFNet network, which added dynamic loss weights and residual fusion block to improve the accuracy of line segmentation. However, these semantic segmentation methods need post-processing to obtain the parking slot, which is time-consuming and inaccurate.

### 2.2. Parking Slot Occupancy Classification

For parking slot occupancy classification, the most common methods are based on distance sensors. Suhr et al. used ultrasonic sensors to determine whether the parking slot is vacant in [21,22,33]. They divided the parking slot into grids and calculated the posterior probability that may be occupied. However, it cannot be applied to these situations where only visual sensors are available. Li et al. [34] adopted the gray histogram to classify the parking slots occupancy. Besides, the stereo vision algorithm was used to identify the existence of small objects by calculating their height. Lee and Seo [35] used slot features, including the Histogram of Gradient (HOG) [36] descriptor and the frequency magnitude feature, via a Support Vector Machine (SVM) [37] to recognize slot occupancy. Similar to [35], Rianto et al. [38] used Local Binary Pattern (LBP) [39] as the method for extracting features of parking slot. Then an SVM classifier was designed to identify the status of the parking slot. However, these conventional feature extraction and classification methods are susceptible to environmental changes.

To further improve the robustness of parking slots occupancy classification under various lighting conditions, Amato et al. [40] proposed the mAlexNet specifically designed for smart cameras to detect parking slot occupancy, which was the first work to employ DCNN on parking lot monitoring images. The mAlexNet was inspired by the AlexNet, where the number of filters and neurons was reduced to improve real-time performance. Nurullayev and Lee [41] designed a generalized parking occupancy classification method for varying images captured from different camera views based on the dilated convolutional neural network. Considering that parking slot occupancy classification is a simple two-category task, a small number of dilated convolutional layers and large kernel sizes were utilized to avoid learning with too deep models. However, these two methods are designed for the monitoring image of the parking lot, where the parking slots can be fully displayed. To make the vacant parking slot detection free from any weather and light conditions, the thermal camera was utilized to detect vehicles based on emitted heat in [42]. The modified Faster RCNN was trained to detect the vacant parking slot. However, when the temperature of the vehicle diminished, the detection accuracy drastically reduced.

## 3. Proposed Method

**Figure 1:**
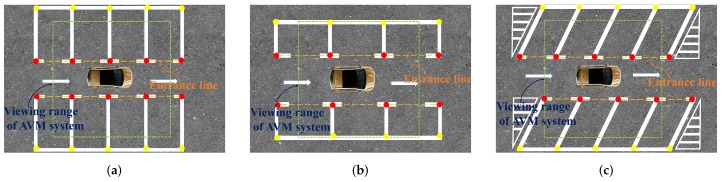
Three typical kinds of parking slots. (**a**) perpendicular parking slots; (**b**) parallel parking slots; (**c**) slanted parking slots. A parking slot consists of four vertices, of which the paired marking points of the entrance line are marked with red dots, and the other two invisible vertices are marked with yellow dots. The entrance lines and the viewing range of an AVM system are also marked out.

**Figure 2:**
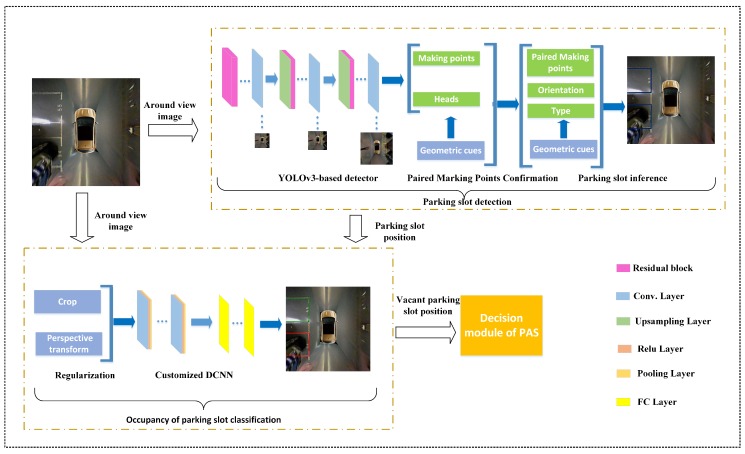
Overview of the VPS-Net, which contains two modules: parking slot detection and occupancy classification. It takes the around view image as input and outputs the position of the vacant parking slot to the decision module of the PAS.

VPS-Net detects various vacant parking slots based on deep learning. As shown in Figure 1, there are three typical kinds of parking slots that VPS-Net can cope with. A parking slot consists of four vertices, two of which are paired marking points of the entrance line, and the other two vertices are usually invisible in the around view image due to limitations of vision. Figure 2 shows the overview of VPS-Net for detecting vacant parking slots. VPS-Net divides vacant parking slot detection into two steps: parking slot detection and occupancy classification, which combines the advantages of a multi-object detection network with a classification network. In the parking slot detection stage, a YOLOv3-based detector is used to detect marking points and parking slot heads simultaneously. Subsequently, geometric cues are used to match paired marking points and determine the orientation of the parking slot. Finally, to obtain the complete parking slot, the two invisible vertices are inferred by the type, orientation, and paired marking points of the parking slot. After the parking slot is detected, its position in the image will be transferred to the occupancy classification part. In the occupancy classification stage, the detected parking slot is first regularized to a uniform size with 120 × 46 pixels, and then a customized DCNN is designed to distinguish whether it is vacant. Once the vacant parking slot is detected, its position will be sent to the decision module of the PAS for further process.

### 3.1. Head and Marking Points of the Parking Slot Detection

**Figure 3:**
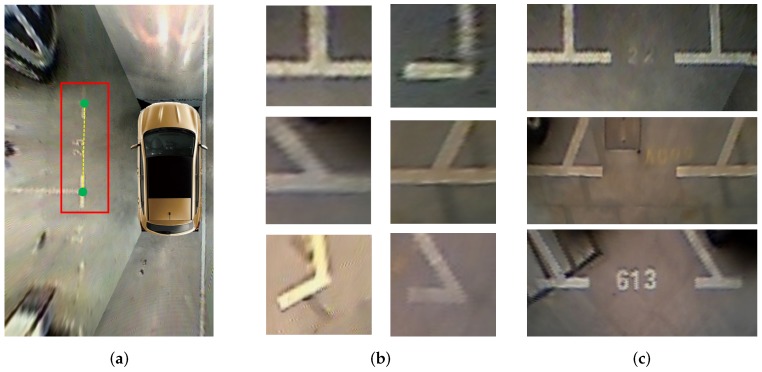
Marking points and parking slot heads. (**a**) shows the geometric relationship between the paired marking points and the parking slot head. Paired marking points are marked with green dots, and the parking slot head is marked with the red rectangle; (**b**) shows a variety of deformations of “T-shaped” or “L-shaped” marking points; (**c**) shows three kinds of the parking slot head belonging to classes “right-angled head”, “obtuse-angled head”, and “acute-angled head” respectively.

**Figure 4:**
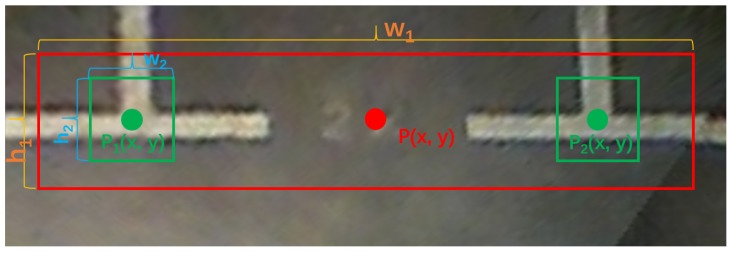
The bounding boxes of the parking slot head and marking points. Each bounding box consists of three parts: coordinates of the center point, width, and height.

As shown in Figure 3, the type of parking slot is determined by its head that contains the paired marking points of the entrance line. Therefore, the parking slot head and marking points detection is the first and most important step of the parking slot detection. PSD_L [10] and DeepPS [11] are two representative marking point-based methods, of which PSD_L uses the machine learning-based detection scheme to detect marking points, and DeepPS utilizes the DCNN-based object detection framework to detect marking points. Despite these two methods that can detect various parking slots effectively, they need a complicated rule-based scheme or a time consuming local image classification to match the paired marking points of the entrance line, resulting in cumbersome steps to infer the complete parking slot. Therefore, different from them, we combine the classification of the parking slot head with the localization of marking points into a multi-target detection problem so that various types of parking slots can be easily inferred based on detection results. For this purpose, we define four kinds of targets, “right-angled head”, “obtuse-angled head”, “acute-angled head”, and “T-shaped” or “L-shaped” marking points respectively. To detect the four types of targets in an around view image, we need to train a detector. Through the literature survey, the multi-object detection based on deep learning could be divided into one-stage method [26,32,43] and two-stage method [44,45,46]. Compared with the two-stage method, the one-stage method processes an image much faster. Considering the real-time requirements of vacant parking slot detection and our detection task is relatively simple, our detector is based on YOLOv3 [26] that is a representative one-state method. To train the YOLOv3-based detector, the training labels including the bounding boxes of parking slot heads and marking points are prepared. As shown in Figure 4, the bounding box of the parking slot head consists of 4 parameters, p(x,y), $w_{1}$, and $h_{1}$, which can be calculated by the coordinates of paired marking points of entrance line by (1)–(3). For each “T-shaped” or “L-shaped” marking point $p_{i}$, its bounding box is a fixed $w_{2}\times h_{2}$ and $p_{i}$ centered rectangle.

(1) $$\begin{array}{c} p\left(x,y\right)=\frac{p_{1}\left(x,y\right)+p_{2}\left(x,y\right)}{2} \end{array}$$

(2) $$\begin{array}{c} w_{1}=\frac{\left|p_{1}\left(x\right)-p_{2}\left(x\right)\right|}{2}+\Delta w \end{array}$$

(3) $$\begin{array}{c} h_{1}=\frac{\left|p_{1}\left(y\right)-p_{2}\left(y\right)\right|}{2}+\Delta h \end{array}$$

where p(x,y) is the center coordinates of the bounding box of the parking slot head. $p_{1}\left(x,y\right)$ and $p_{2}\left(x,y\right)$ are the coordinates of paired marking points of the entrance line. $w_{1}$ is the width of the bounding box, $h_{1}$ is the height of the bounding box. Δw and Δh are hyperparameters that control the width and height of the bounding box.

In the implementation process, we use the Darknet-53 pre-trained on ImageNet [47] as the feature extractor of YOLOv3-based detector and then fine-tune the ps2.0 dataset [11]. In the process of fine-tuning, the batch size is 32, the image is scaled to 416×416, the anchors are modified for ps2.0 dataset to [(10, 13), (28, 42), (33, 23), (30, 61), (62, 45), (61, 199), (126, 87), (156, 198)], and the learning rate starts from 0.0001 and is decayed by 10 every 45,000 steps. The Adam optimizer is used with the proposed optimization setting in [48] with [$\beta_{1}$, $\beta_{2}$, ε] = [0.9, 0.999, $10^{-8}$]. Data augmentation is performed during the training process. We flip the image and the corresponding bounding box with a 50% probability level. We also add color augmentations with a 50% chance, including random saturation with [1.0, 1.5], and random exposure with [1.0, 1.5] in the HSV color space.

### 3.2. Paired Marking Points of Entrance Line Confirmation

**Figure 5:**
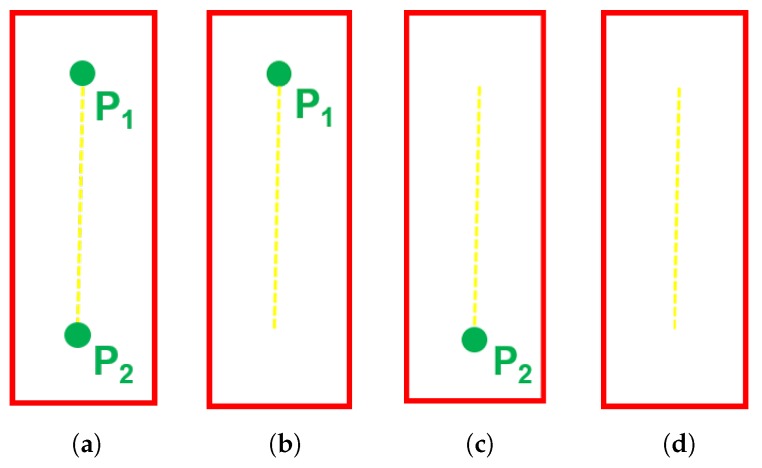
The relationship between two marking points $p_{1}$, $p_{2}$ and the bounding box of the parking slot head B. (**a**) shows $p_{1}\subseteq B$ and $p_{2}\subseteq B$; (**b**) shows $p_{1}\subseteq B$ and $p_{2}⊄B$; (**c**) shows $p_{1}⊄B$ and $p_{2}\subseteq B\;$; (**d**) shows $p_{1}⊄B$ and $p_{2}⊄B$.

After the detector detects marking points and parking slot heads, we design the Algorithm 1 using the detection results and geometric cues to match paired marking points. Suppose that $p_{1}$, $p_{2}$ are two marking points and B is the bounding box of the parking slot head. As shown in Figure 5, the relationship between $p_{1}$, $p_{2}$ and B can be divided into four types. If both $p_{1}$ and $p_{2}$ are contained in B, then $p_{1}$ and $p_{2}$ are paired marking points. If $p_{1}$ or $p_{2}$ is contained in B and the object confidence of B is greater than 95%, then $p_{i}$ and ${p_{i}}^{\prime }$ are paired marking points. ${p_{i}}^{\prime }$ can be calculated by Equation (4).

(4) $$\begin{array}{c} {p_{i}}^{\prime }=∼b_{j}\left(j≜\min∥p_{i}-b_{j}∥\right)-\left(\Delta w,\;\Delta h\right) \end{array}$$

where $b_{j}$ is one of the four vertices of B and $∼b_{j}$ represents the diagonal vertex of $b_{j}$. Δw and Δh are hyperparameters that control the width and height of B.
**Algorithm 1** Rules of paired marking points confirmation**Input:**   Two sets B and P, comprising all bounding boxes and marking points in an around view image, respectively.**Output:**  Paired marking points.
1:**for**B in B **do**2:  **for** p in P **do**3:    Count the number N of p in B4:  **end for**5:  **if** N = 2 **then**6:    $p_{1}$ and $p_{2}$ are paired marking points7:  **end if**8:  **if** N = 1 and the confidence of B > 95% **then**9:    Step 1: Calculate the other marking point ${p_{2}}^{\prime }$ using Equation (4)10:    Step 2: $p_{1}$ and ${p_{2}}^{\prime }$ are paired marking points11:  **end if**12:  **if** N = 0 and the confidence of B > 98% **then**13:    Step 1: Calculate the NAIV of the four vertex regions of B using Equation (5)14:    Step 2: The largest NAIV set of diagonal vertices ${p_{1}}^{\prime }$ and ${p_{2}}^{\prime }$ are paired marking points15:  **end if**16:  **if** N > 2 **then**17:    Two points $p_{1}$ and $p_{2}$ that is the closest to the diagonal vertices of B are paired marking points18:  **end if**19: **end for**

If neither $p_{1}$ nor $p_{2}$ is contained in the B and the object confidence of B is greater than 98%, then the normalized average intensity values (NAIV) [19] of the four vertex regions are calculated by Equation (5) and the largest NAIV set of diagonal vertices is selected as the paired parking points. This is because marking points are much brighter than the ground plane and the pixels near marking points tend to have greater intensity [12].

(5) $$\begin{array}{c} NAIV_{i}=\frac{1}{MAX(I)}\left\{\frac{1}{N}\sum_{x,y\in R_{i}}I(x,y)\right\} \end{array}$$

where $NAIV_{i}$ is the NAIV of the vertex i-centered region $R_{i}$ of fixed size 10 × 10 pixels. MAX(I) is the maximum intensity value of the image *I*. *N* and (x,y) are the number of pixels in the region $R_{i}$ and their locations in the x-axis and y-axis.

If there are more than two marking points in the bounding B, the two marking points that are the closest to the diagonal vertices of B are paired marking points. After that, the type of parking slot can be determined by the distance between the paired marking points and the type of parking slot head. When the head of the parking slot is classified as a “right-angled head”, if the distance is less than *t*, it is considered as a perpendicular parking slot, otherwise, it is a parallel parking slot. If the head of the parking slot is classified as an “obtuse-angled head” or an “acute-angled head”, it is considered as a slanted parking slot.

### 3.3. Complete Parking Slot Inference

**Figure 6:**
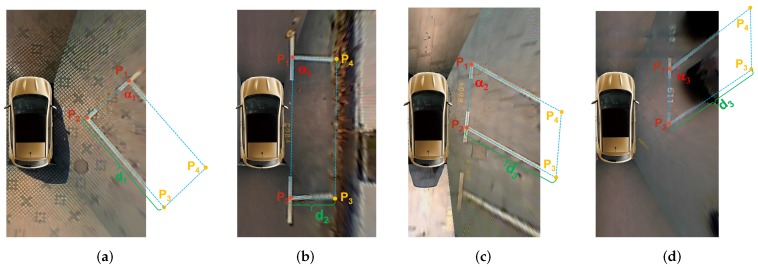
Complete parking slot inference. (**a**–**d**) are the perpendicular parking slot, the parallel parking slot, the slanted parking with an acute angle, and the slanted parking with an obtuse angle respectively. Their depth is $d_{1}$, $d_{2}$ and $d_{3}$ respectively, and their parking angle is $\alpha_{1}$, $\alpha_{2}$ and $\alpha_{3}$ respectively. $p_{1}$, $p_{2}$ are two visible paired marking points, and $p_{3}$, $p_{4}$ are two invisible vertices.

In around view images, most of parking slots are not fully displayed, so we need to infer the complete parking slot based on the geometry cues and prior knowledge of the parking slot. As shown in Figure 6, the parking slot is presented by four vertices, of which $p_{1}$, $p_{2}$ are paired marking points, and $p_{3}$, $p_{4}$ are two invisible vertices. The two invisible vertices of the parking slot $p_{3}$, $p_{4}$, can be calculated via Equations (6) and (7).

(6) $$\begin{array}{c} p_{3}=\left[\begin{array}{cc} \cos\alpha_{i} & \sin\alpha_{i}\\ -\sin\alpha_{i} & \cos\alpha_{i} \end{array}\right]\frac{\vec{p_{1}p_{2}}}{∥\vec{p_{1}p_{2}}∥}d_{i}+p_{2} \end{array}$$

(7) $$\begin{array}{c} p_{4}=\left[\begin{array}{cc} \cos\alpha_{i} & \sin\alpha_{i}\\ -\sin\alpha_{i} & \cos\alpha_{i} \end{array}\right]\frac{\vec{p_{1}p_{2}}}{∥\vec{p_{1}p_{2}}∥}d_{i}+p_{1} \end{array}$$

where $\alpha_{i}$ and $d_{i}$ are the parking angle and the depth of the parking slot, respectively.

The parking angle $\alpha_{i}$ can be determined by the type of the parking slot head and the orientation of the parking slot. The depth $d_{i}$ can be choosen as different values according to the type of the parking slot. For the perpendicular parking slot or the parallel parking slot, $\alpha_{i}=\pm \alpha_{1}$ and $d_{i}\;=\;d_{1}$ or $d_{i}=d_{2}$. For the slanted parking slot with an acute angle, $\alpha_{i}=\pm \alpha_{2}$ and $d_{i}=d_{3}$. For the slanted parking slot with an obtuse angle, $\alpha_{i}=\pm \alpha_{3}$ and $d_{i}=d_{3}$. When the four vertices of the parking slot are arranged clockwise, $\alpha_{i}$ is positive. Otherwise, it is negative.

**Figure 7:**
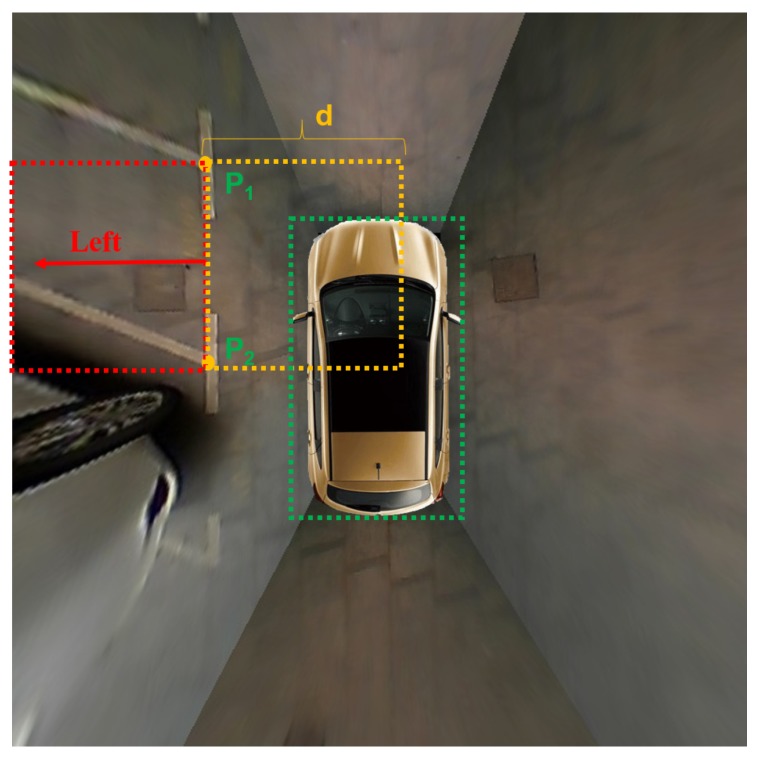
The orientation of the parking slot when the vehicle is around it. Two rectangular boxes formed by the entrance line with a depth *d* are marked with red and orange dotted lines. The rectangular box formed by the car model is marked with gree dotted lines. The red arrow indicates the orientation of the parking slot.

**Figure 8:**
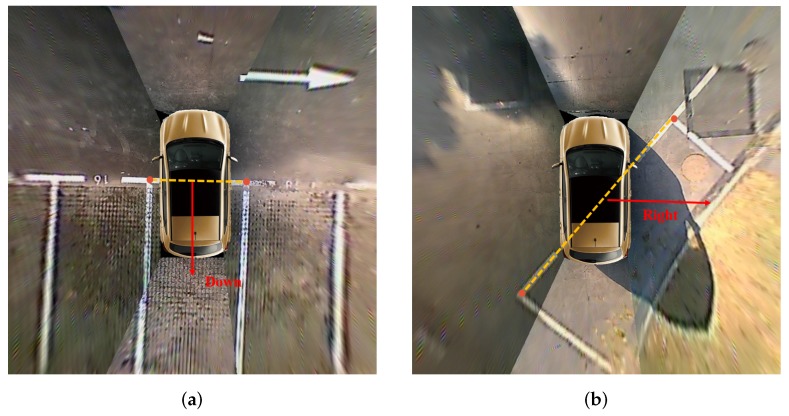
The orientation of the parking slot when the vehicle is parking into it. (**a**) shows the orientation of the vertical parking slot. (**b**) shows the orientation of the parallel parking slot. The red arrow indicates the orientation of the parking slot. The yellow dotted line indicates the entrance line.

Since the orientation of the parking slot determines whether the four vertices of the parking slot are arranged clockwise or counterclockwise, it should be identified through geometric cues. For the parking slot around the vehicle, the entrance line does not intersect the rectangular box formed by the car model. Therefore, as shown in Figure 7, the orientation of the parking slot can be determined according to the IOU between the rectangular box formed by the entrance line and the rectangular box formed by the car model. The IOU can be calculated by Equation (8). For the vehicle parking into the parking slot, the entrance line intersects the rectangular box formed by the car model, as shown in Figure 8. If it is the vertical parking slot or the slanted parking slot, the orientation of the parking slot is considered to be the downward direction. If it is the parallel parking slot and the slope of the entrance line is positive, the orientation of the parking slot is the right direction. Otherwise, the orientation of the parking slot is the left direction.

(8) $$\begin{array}{c} IOU=\frac{Area1\cap Area2}{Area1\cup Area2} \end{array}$$

where Area1 is the the rectangular box formed by the entrance line and Area2 is the the rectangular box formed by the car model.

### 3.4. Parking Slot Occupancy Classification

**Figure 9:**
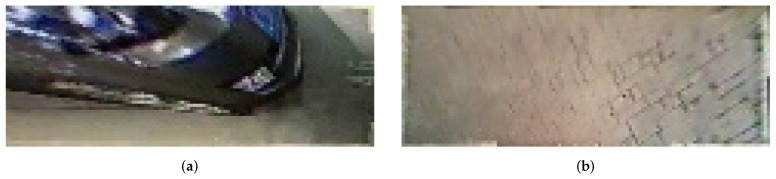
Training samples for vacant parking slot classification. (**a**) a negative sample: a non-vacant regularized parking slot. (**b**) a positive sample: a vacant regularized parking slot.

This is the last step of VPS-Net. After the complete parking slot is inferred, its position in the image will be transferred to this part to distinguish whether it is vacant. Since parking slots in an around view image are vary in size, a regularized form is required to maximize classification performance. As shown in Figure 9, the parking slot is cut and warped to a uniform size with 120 × 46 pixels according to its position in the image. The perspective transform technique is used to implement this warping process. The four boundary points of the parking slot in an image serve as source points, whereas the destination points are the four vertices of the fixed rectangle with 120 × 46 pixels. In this way, we can obtain a series of labeled images, which are divided into positive samples and negative samples. The positive samples are vacant parking slots, and the negative samples are non-vacant parking slots. We perform a 180${}^{\circ }$ rotation transformation to further increase the number of training samples.

After obtaining a large number of training samples, the data-driven learning-based methods can be utilized to classify whether the parking slot is vacant, which can be divided into the classical machine learning-based methods and the deep learning-based methods. The classical machine learning-based methods first use artificially designed features, such as HOG descriptors [36] or LBP descriptors [38], to represent the regularized parking slot samples, and then train an SVM classifier model [37] to implement the occupancy classification. The deep learning-based methods directly use regularized parking slot samples to train DCNN models, such as AlexNet [49], VggNet [50], ResNet [51], and MobileNet [52], to achieve the occupancy classification. Although the classical machine learning-based methods are easy to be deployed and implemented, it is susceptible to lighting variations and diverse circumstances. In addition, considering the existing DCNN models have specific requirements for the input size of the image, and their structure is relatively complicated, we specially design a customized DCNN model based on AlexNet for the parking slot occupancy classification. As shown in Table 1, it is a detailed description of our customized DCNN model. Compared with AlexNet, the number of filters of convolutional layers and the number of neurons of fully connected layers is reduced to decrease the computational complexity. Besides, the customized DCNN model takes a 120 × 46 RGB image as input, and the kernel size of filters is adjusted according to the size of the input image, which significantly reduces the parameters. In the implementation process, the customized DCNN model is first pre-trained on the ImageNet [47], and then is fine-tuned in our training samples, including 12,772 positive samples and 5066 negative samples. The choices of optimizer, the learning rate setting and the data augmentation are the same as when the YOLOv3-based detector is trained.

It is worth noting that we can easily get the transformation matrix from the around view image coordinate system to the vehicle-centered world coordinate system in the around view image synthesis process. Therefore, once the vacant parking slot in the around view image is detected, its physical position can be calculated using the transformation matrix, which will be sent to the decision module of the PAS. Then, the PAS plans an optimal path to the vacant parking space based on its position. Finally, the parking guidance by a graphical user interface or path tracking by active steering is implemented by the PAS.

## 4. Experiments and Results

### 4.1. Experiments Setup

#### 4.1.1. Datasets

To verify the performance of VPS-Net, we conduct experiments in the largest around view images dataset called ps2.0 [11]. The images in the ps2.0 dataset are collected from various environmental conditions through an AVM system with four low-cost fish-eye cameras equipped on a SAIC Roewe E50 electric car. It contains 12,165 around view images with 600 × 600 pixels corresponding to a 10 m × 10 m physical plane region, of which 9827 images are training samples, and 2338 images are testing samples. To evaluate the performance of VPS-Net under different environmental conditions, the test dataset is divided into the following six categories: “indoor”, “outdoor daylight”, “outdoor street light”, “outdoor shadow”, “outdoor rainy”, and “slanted". However, the ps2.0 dataset is only designed for parking slot detection, and it does not include the occupancy status of the parking slot. Therefore, we mark the type of parking slot in each image to generate a new dataset for occupancy classification. It needs to be noted that there may be multiple parking slots in an around view image. Consequently, for the training dataset of occupancy classification, a total of 17,838 parking slot samples are obtained, of which 12,772 samples are vacant parking slots, and 5066 samples are non-vacant parking slots. For the testing dataset of occupancy classification, a total of 2145 parking slot samples are obtained, of which 1596 samples are vacant parking slots, and 549 samples are non-vacant parking slots. All these samples are cut and warped to a uniform size with 120 × 46 pixels.

**Figure 10:**
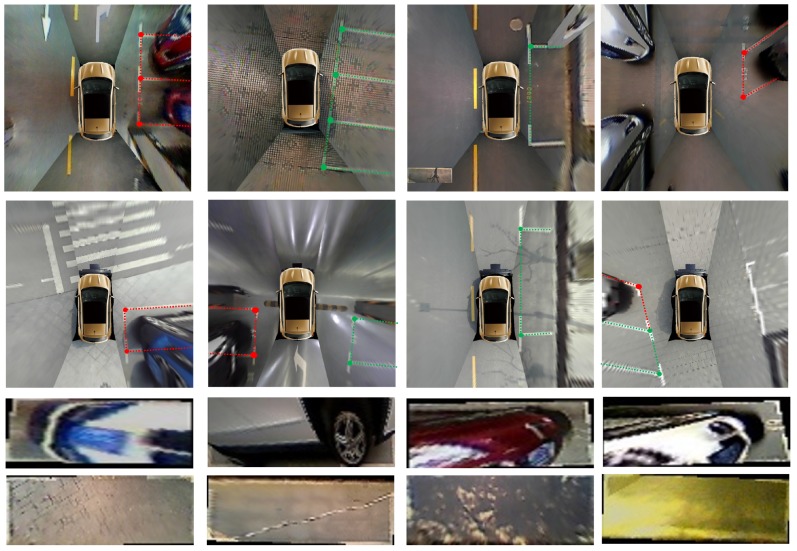
Cases of datasets used in evaluation. Rows 1 and 2 are the annotation information that was labeled for ps2.0 and PSV datasets. The green indicates the vacant parking slot. The red indicates the non-vacant parking slot. Rows 3 and 4 are parking slot samples that were cut and warped according to the annotation information.

Furthermore, the PSV dataset [24] is used to verify the generalizability of the VPS-Net. There are a total of 4249 around view images in the PSV dataset, of which 1274 images are for testing. However, the PSV dataset only contains segmentation labels of parking slots. Therefore, we add the new annotation to the PSV dataset. Some cases of these datasets are shown in Figure 10. To facilitate future researchers, the annotations for vacant parking slots of ps2.0 and PSV datasets have been made publicly available at https://github.com/weili1457355863/VPS-Net.

#### 4.1.2. Experiment Settings

There are some hyper-parameters that need to be set for the VPS-Net. All hyper-parameters correspond to a resolution of 600×600 pixels and are summarized in Table 2. Their detailed meaning can refer to Section 3. Δw and Δh are hyperparameters that control the width and height of the bounding box of the parking slot head. $w_{2}$ and $h_{2}$ are the width and height of the bounding box of the marking point. $\alpha_{1}$, $\alpha_{2}$, and $\alpha_{3}$ are the parking angle of parking slots. $d_{1}$, $d_{2}$, and $d_{3}$ are the depth of the perpendicular parking slot, parallel parking slot, and slanted parking slot. *t* is the threshold for distinguishing between the perpendicular parking slot and the parallel parking slot. *d* is the depth for determining the orientation of the parking slot.

We implement the VPS-Net using the publicly available Pytorch framework [53] in Ubuntu 16.04. All experiments are conducted in a server with the Intel Core i9-7900X CPU @3.30GHz×10, two Nvidia Titan Xp GPU cards, and 32G RAM.

### 4.2. Heads and Marking Points Detection Performance

**Figure 11:**
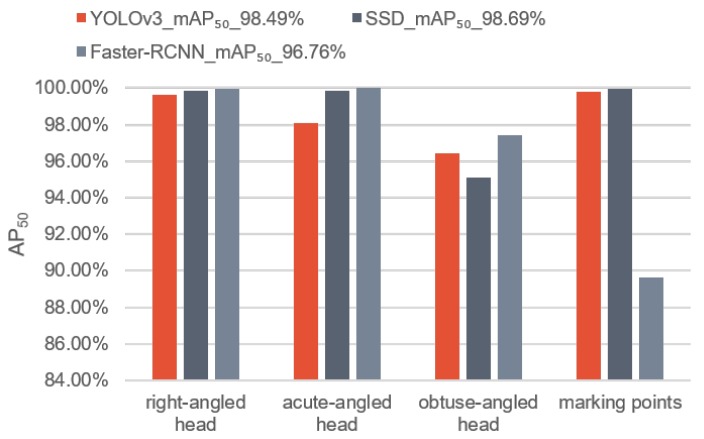
AP${}_{50}$ histograms by three kinds of DCNN-based detectors.

As the first step of VPS-Net, the detection performance of the three kinds of parking slot heads and marking points is important. In this experiment, we compare the YOLOv3-based detector with two SOTA object detection networks, SSD [43] and Faster-RCNN [46]. Both SSD and Faster-RCNN use the VGG16 [50] as the backbone, and the YOLOv3-based detector uses the Darknet-53 as the backbone. The evaluation is performed on the test dataset of ps2.0 [11]. To compare the different models, we draw the average precision with the IOU threshold set to 50% (AP${}_{50}$) histograms of the different objects of each model, as shown in Figure 11. We use mean average precision with the IOU threshold set to 50% (mAP${}_{50}$) to summarize the performance of the model, which can be calculated via (9)–(13)

(9) $$\begin{array}{c} \rho =\frac{true\;positives}{true\;positives+false\;positives} \end{array}$$

(10) $$\begin{array}{c} r=\frac{true\;positives}{true\;positives+false\;negatives} \end{array}$$

(11) $$\begin{array}{c} \rho inter\;p\left(r_{n+1}\right)=\max_{\tilde{r}:\tilde{r}\geq r_{n+1}}\;\rho \left(\tilde{r}\right) \end{array}$$

(12) $$\begin{array}{c} AP=\sum_{r=0}^{1}\left(r_{n+1}-r_{n}\right)\rho inter\;p\left(r_{n+1}\right) \end{array}$$

(13) $$\begin{array}{c} mAP=\frac{1}{N}AP \end{array}$$

where ρ is the precision rate and *r* is the recall rate. $\rho \left(\tilde{r}\right)$ is the measured precision rate at the recall rate $\tilde{r}$ and $\rho inter\;p\left(r_{n+1}\right)$ takes the maximum precision whose recall rate is greater or equal than $r_{n+1}$. *N* is the total number of objects.

**Figure 12:**
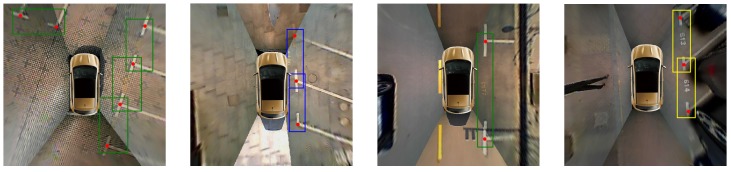
Detection results by YOLOv3-based detector. The green bounding box indicates the “right-angled head”. The blue bounding box indicates the “acute-angled head”. The yellow bounding box indicates the “obtuse-angled head”. The red dot indicates the “marking point”.

As shown in Figure 11, three DCNN-based multi-object detection models all achieve good results, of which the YOLOv3-based detector and SDD [43] are more accurate than Faster-RCNN [46] for marking points. Since the position of the parking slot in the image is determined by marking points, the localization accuracy of marking points is very important. We calculate the localization error using the mean and the standard deviation for all true positives of marking points. Besides, considering that the real-time requirement of vacant parking slot recognition is relatively high, we also test the running time of these three methods to detect an image. As shown in Table 3, the YOLOv3-based detector locates marking points more accurately and takes less time to process an image than the other two SOTA models. Therefore, we take the YOLOv3-based detector to detect marking points and parking slot heads. These results in Figure 12 show that the YOLOv3-based detector performs well. It can accurately detect three kinds of parking slot heads and marking points in various conditions.

### 4.3. Parking Slot Detection and Occupancy Classification Performance

**Figure 13:**
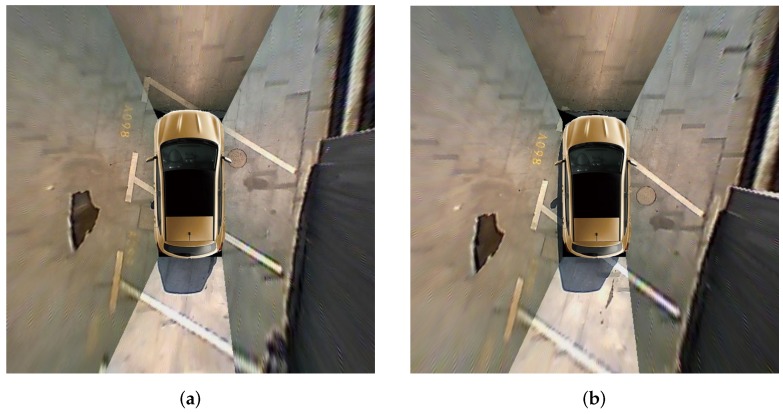
(**a**,**b**) show representative images in the ps2.0 test dataset where the vehicle is across parking slots.

VPS-Net divides the vacant parking slot detection into two steps: parking slot detection and occupancy classification. In this experiment, we evaluate them respectively. For parking slot detection, we evaluate it using the precision rate, the recall rate and the localization error in the ps2.0 dataset and compare it with several SOTA methods in this field including PSD_L [10] and DeepPS [11]. Table 4 lists the detection performance of these methods. In this table, #GT, #TP, and #FP indicate the number of ground truths, true positives, and false negatives in the test dataset. The VPS-Net outperforms the machine learning method called PSD_L by 1.03% and 14.37% in terms of precision rate and recall rate, respectively. Besides, our method gives a 0.19% lower precision rate but a 0.64% higher recall rate than the DCNN-based method called DeepPS. This is because DeepPS produces no parking slot when it cannot detect marking points, which means the number of false positives is lower than VPS-Net, and there are four images in the test set where the vehicle is across in the parking slot, as shown in Figure 13. This case violates our principle of estimating the orientation of the parking slot based on geometric cues, and it is meaningless for the PAS. Therefore, we also evaluate the performance of VPS-Net and DeepPS after removing these images. Under these conditions, VPS-Net gives a 0.14% higher precision rate and a 0.92% higher recall rate than DeepPS. To further verify the parking slot detection robustness of VPS-Net in various conditions, we evaluate it in the sub-test sets of ps2.0, and the results are summarized in Table 5. ρ and *r* indicate the precision rate and the recall rate, respectively. VPS-Net can detect parking slots robustly under various environmental conditions. Additionally, Table 6 lists the localization error of two visible paired marking points for all true positives of parking slots. VPS-Net has higher localization accuracy for parking slots than other learning-based methods. In general, VPS-Net outperforms these two previous methods for parking slot detection.

**Figure 14:**
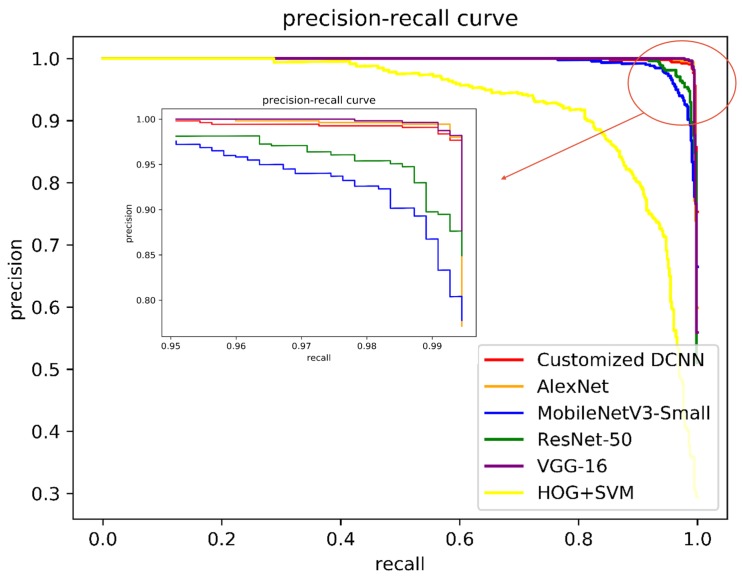
Precision-recall curves of different methods for parking slot occupancy classification.

For occupancy classification, we evaluate the performance of the customized DCNN in the self-annotated testing dataset and compare it with the conventional feature extraction and classification technique (HOG+SVM) [37] and some SOTA networks including AlexNet [49], VGG-16 [50], ResNet-50 [51], and MobileNetV3 [52], in terms of the classification accuracy, the running time, the model size, and the precision-recall curve. As shown in Figure 14 and Table 7, all DCNN models outperform the conventional feature extraction and classification technique (HOG+SVM). Besides, compared with other DCNN models, our customized DCNN model not only achieves comparable accuracy but also consumes less time to process an image and has fewer parameters.

### 4.4. Overall Performance and Generalizability of VPS-Net

**Figure 15:**
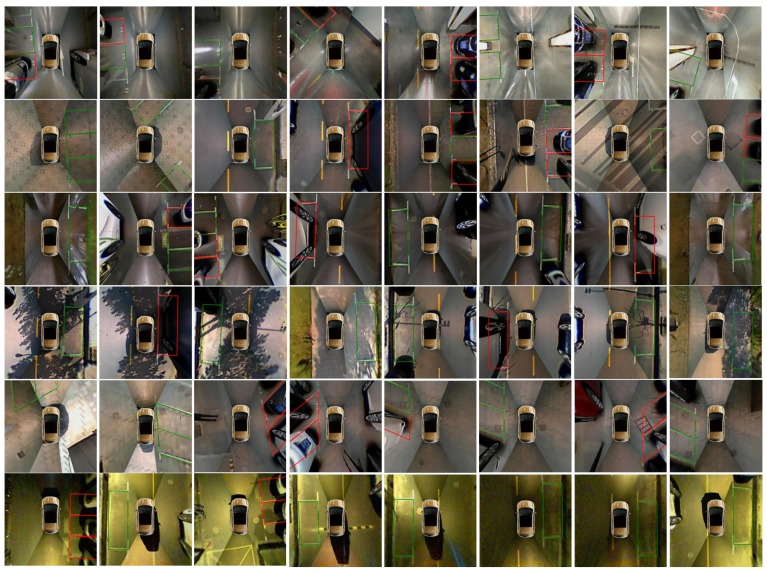
VPS-Net detection results. Green indicates the vacant parking slot. Red indicates the non-vacant parking slot. Different rows show three kinds of parking slots in various imaging conditions like ’indoor’, ’outdoor daylight’, ’outdoor rainy’, ’outdoor shadow’, ’outdoor slanted’, ’outdoor street light’ respectively.

We evaluate the overall performance of the VPS-Net in the modified ps2.0 with no vehicle across the parking slot. Table 8 lists the overall performance of VPS-Net in the ps2.0 test set. The VPS-Net achieves the precision rate of 99.63% and the recall rate of 99.31% toward vacant parking slot detection. Besides, the VPS-Net takes about a total of 20.5 ms to process an image, meeting the real-time requirements of PAS. Among them, marking points and the parking slot head detection takes 18 ms, complete parking slot inference takes 0.5 ms, and parking slot occupancy classification takes 2 ms. As shown in Figure 15, three typical kinds of parking slots under different imaging conditions are marked, with the green indicating a vacant parking slot and the red indicating a non-vacant parking slot. Despite the visual environment is very complicated, such as reflections of light on the ground surface, shadows of trees or vehicles, diverse ground materials, dim street light and limitations of vision, VPS-Net can detect different types of parking slots accurately and classify whether it is vacant robustly.

**Figure 16:**
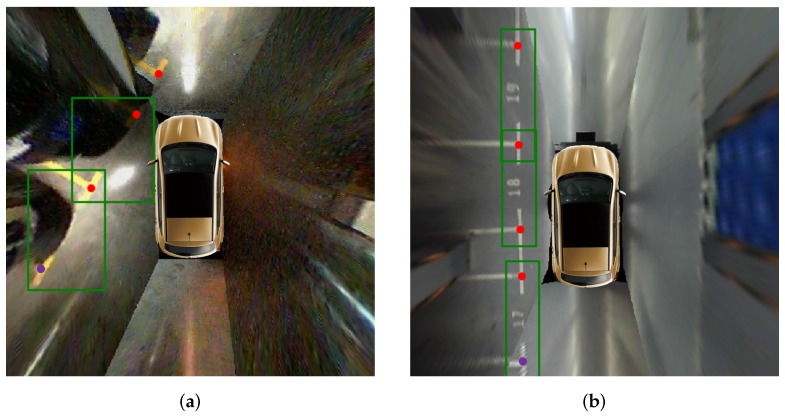
Representative images with the degraded image quality of marking points in the PSV dataset. (**a**) shows the marking point is far from cameras. (**b**) shows the marking point is on the stitching lines. The green bounding box indicates the parking slot head. The red dot indicates the detected marking point, and the purple dot indicates the inferred marking point based on the parking slot head.

Additionally, we verify the generalization performance of the VPS-Net in the PSV dataset [24]. It needs to be noted that we do not train the VPS-Net in the PSV dataset, and there are several images with 1000 × 1000 pixels, which have a large domain gap with the ps2.0 dataset. Table 9 lists the detection results of DeepPS [11] and VPS-Net. VPS-Net obtains a 0.86% higher precision rate and a 6.97% higher recall rate than DeepPS toward parking slot detection, which means VPS-Net can detect more true parking slots for new images. Besides, Algorithm 1 of VPS-Net improves the recall rate by 1.51%. This is because the image quality of marking points located far from camera or stitching lines is degraded for some images, as shown in Figure 16, but VPS-Net can match paired marking points based on the detected parking slot head in this case. Moreover, for vacant parking slot detection, the precision rate of 95.58% and the recall rate of 93.66% are achieved by VPS-Net. On average, the results show that VPS-Net has a satisfying generalization performance.

## 5. Discussion

The above experimental results reveal that VPS-Net can quickly and effectively detect various vacant parking slots by combining a one-stage detector with a customized DCNN. For parking slot heads and marking points detection, since we modify the anchors of YOLOv3 and train it specifically for this task, it achieves higher detection accuracy and less time than the other two SOTA object detection networks including SSD [43] and Faster-RCNN [46]. For parking slot detection, VPS-Net outperforms previous methods such as PSD_L [10] and DeepPS [11]. This is because the YOLOv3-based detector as used in VPS-Net has higher accuracy of detection and localization for marking points detection than ACF + Boosting as used in PSD_L and YOLOv2-based detector as used in DeepPS. Besides, we design a simple and efficient algorithm based on detection results and geometric cues to match paired marking points, which can still match the paired marking points based on the detected parking slot head, even if some of the marking points are not detected. For parking slot occupancy classification, we specially design a customized DCNN model based on AlexNet [49]. Since the input image is warped to 120 × 46 pixels, we adjust the convolution kernel size of the first two convolution layers so that the convolution kernel has a more appropriate receptive field to extract more suitable features. Moreover, as our task is relatively simple, we remove a convolutional layer and a fully connected layer and reduce the number of output channels per layer, which reduces the running time and parameters of the model. Therefore, our customized DCNN model not only achieves comparable accuracy but also consumes less time to process an image.

However, VPS-Net currently is not perfect. When the imaging conditions are particularly poor, VPS-Net may miss some parking slots because the marking points of the entrance line are not salient, and the confidence of the parking slot head is less than the threshold. Occasionally, VPS-Net will incorrectly classify whether a parking slot is vacant. This is because the parking slot is too far from the vehicle, and only a small part is in the around view image. Apart from that, some environmental conditions are not included in the training dataset, like snowy conditions and foggy conditions. Therefore, there is still some room for improvement in future research: (1) We will establish a more extensive scale dataset. (2) To make vacant parking slot detection more straightforward, we will explore a single neural network to detect the parking slot and classify the occupancy status simultaneously. (3) We will comprehensively utilize the detection results of multiple frames to make parking slot detection more accurate. (4) During the parking process, sometimes the parking slot head in the around view image is incomplete, which will result in failing to detect the parking slot. Therefore, we will continue to study the tracking of the paired marking points during parking, so as to obtain better performance of parking slot recognition and localization.

## 6. Conclusions

The detection of vacant parking slots is the first and significant step of PAS. This paper proposes a DCNN-based vacant parking slot recognition and localization method, namely VPS-Net. VPS-Net converts the vacant parking slot detection into a combination of multi-object detection and classification problems based on DCNN to improve its performance in the complicated visual environment. For the parking slot detection, we combine the classification of the parking slot with the localization of marking points using a YOLOv3-based detector so that the parking slot can be directly inferred. For the occupancy classification, we design a customized classification network so that the parking-availability classification can be achieved reliably. After verification in the largest around view images dataset ps2.0 [11], the VPS-Net can detect various vacant parking slots robustly with the precision rate of 99.63% and the recall rate of 99.31% and locate the two visible paired marking points of the parking slot accurately with the localization error of 1.03 ± 0.64 pixels. Moreover, VPS-Net has a satisfying generalization performance through testing in the PSV dataset [24].

## Figures and Tables

**Table 1:** Detailed description of the customized DCNN for parking slot occupancy classification.

Layer Name	Kernel	Padding	Stride	Output (CxHxW)
Input	-	-	-	3 × 46 × 120
Conv1	[3, 9]	[0, 0]	[1, 2]	40 × 44 × 56
Maxpool1	[3, 3]	[0, 0]	[2, 2]	40 × 21 × 27
Conv2	[3, 5]	[1, 0]	[1, 1]	80 × 21 × 23
Maxpool2	[3, 3]	[1, 0]	[2, 2]	80 × 11 × 11
Conv3	[3, 3]	[1, 1]	[1, 1]	120 × 11 × 11
Conv4	[3, 3]	[1, 1]	[1, 1]	160 × 11 × 11
Maxpool2	[3, 3]	[0, 0]	[2, 2]	160 × 5 × 5
Fc1	-	-	-	512 × 1 × 1
Fc1	-	-	-	2 × 1 × 1

**Table 2:** Setting for hyper-parameters of VPS-Net.

Parameter	Value (pixels)	Parameter	Value (pixels)
Δw	48	$\alpha_{2}$	67
Δh	44	$\alpha_{3}$	129
$w_{2}$	40	$d_{1}$	250
$h_{2}$	60	$d_{2}$	125
*t*	190	$d_{3}$	240
$\alpha_{1}$	90	*d*	250

**Table 3:** Localization error of marking points and running time of three kinds of DCNN-based detector.

Method	Localization Error (in pixel)	Localization Error (in cm)	Running Time (ms)
Faster-RCNN [46]	3.67 ± 2.32	6.12 ± 3.87	45
SSD [43]	1.51 ± 1.17	2.52 ± 1.95	26
YOLOv3-based	**1.03 ± 0.65**	**1.72 ± 1.09**	**18**

**Table 4:** Parking slot detection performance of different methods in the ps2.0 test set.

Method	#GT	#TP	#FP	Precision Rate	Recall Rate
PSD_L [10]	2173	1845	27	98.55%	84.89%
DeepPS [11]	2173	2143	5	99.77%	98.62%
VPS-Net	2173	2157	9	99.58%	99.26%
DeepPS (no across)	2166	2137	5	99.77%	98.66%
VPS-Net (no across)	2166	2157	2	**99.91**%	**99.58**%

**Table 5:** Parking slot detection performance of different methods in the ps2.0 sub-test sets.

Sub-Test Set	DeepPS [11]	VPS-Net	VPS-Net (No Across)
Indoor	ρ**: 100.00%**; *r*: 97.67%	ρ: 99.71% *r*: 98.54%	ρ: 99.71%; *r***: 98.54%**
Outdoor normal	ρ: 99.87%; *r*: 98.85%	ρ: 100.00%; *r*: 99.74%	ρ **: 100.00%;** *r* **: 99.74%**
Street light	ρ: 100.00%; *r*: 100.00%	ρ: 100.00%; *r*: 100.00%	ρ **: 100.00%;** *r* **: 100.00%**
Outdoor shadow	ρ: 99.86%; *r*: 99.14%	ρ: 100.00%; *r*: 99.86%	ρ **: 100.00%;** *r* **: 99.86%**
Outdoor rainy	ρ: 100.00%; *r*: 99.42%	ρ: 100.00%; *r*: 100.00%	ρ **: 100.00%;** *r* **: 100.00%**
Slanted	ρ: 96.15%; *r*: 92.59 %	ρ: 90.12%; *r*: 90.12%	ρ **: 98.65%;** *r* **: 98.65%**

**Table 6:** Localization error of parking slots of different methods in the ps2.0 test set.

Method	Localization Error (in pixel)	Localization Error (in cm)
PSD_L [10]	3.64 ± 1.85	6.07 ± 3.09
DeepPS [11]	1.55 ± 1.04	2.58 ± 1.74
VPS-Net	**1.03 ± 0.64**	**1.72 ± 1.07**

**Table 7:** Performance of parking slot occupancy classification of different methods in the self-annotated testing dataset.

DCNN Model	Accuracy	Running Time (ms)	Model Size (MB)
HOG+SVM [37]	92.54%	2.13	**0.04**
AlexNet [49]	**99.67%**	1.75	228.1
VGG-16 [50]	99.62%	2.15	537.1
ResNet-50 [51]	98.55%	5.10	44.8
MobileNetV3-Small [52]	98.55%	6.21	5.1
Customized DCNN	99.48%	**0.81**	9.4

**Table 8:** Overall performance of VPS-Net in the ps2.0 test set.

Step	Running Time (ms)	Precision Rate	Recall Rate
marking points and heads detection	18	-	-
complete parking slot inference	0.5	99.91%	99.58%
parking slot occupancy classification	2	99.86%	99.62%
total	20.5	99.63%	99.31%

**Table 9:** Parking slot detection performance of different methods in the PSV test set.

Method	#GT	#TP	#FP	Precision Rate	Recall Rate
DeepPS [11]	1593	1396	63	95.68%	87.63%
VPS-Net (no Algorithm 1)	1593	1483	50	**96.73%**	93.09%
VPS-Net	1593	1507	54	96.54%	**94.60%**

## Abbreviations

The following abbreviations are used in this manuscript:

PAS	Park assist system
AVM	Around view monitor
DCNN	Deep convolutional neural network
SOTA	State of the art
